# Supplementary material for: Dietary Patterns and New-Onset Type 2 Diabetes Mellitus in Evacuees after the Great East Japan Earthquake: A 7-Year Longitudinal Analysis in the Fukushima Health Management Survey
Source: Nutrients. 2022 Nov 17;14(22):4872. doi: 10.3390/nu14224872 (PMC9694161; doi:10.3390/nu14224872)
Supplement: Supplementary file 1 [file nutrients-14-04872-s001.zip › nutrients-1901532-supplementary.pdf]

nutrients

MDPI

Table S1. Factor loadings of dietary patterns identified in participants at baseline in 2011, FHMS[a].

	Men			Women		
Food Item	Typical	Juice	Meat	Typical	Juice	Meat
Boiled bean	**0.414**	**0.397**	0.058	**0.435**	**0.324**	-0.011
Fermented bean	**0.481**	0.123	-0.147	**0.513**	0.130	-0.120
Fish	**0.534**	0.059	0.204	**0.575**	0.060	0.117
Fruit	**0.514**	**0.402**	-0.039	**0.535**	**0.341**	-0.099
Green vegetable	**0.694**	0.185	0.205	**0.670**	0.135	0.151
Miso soup	**0.645**	-0.094	-0.119	**0.616**	-0.161	-0.069
Red/yellow vegetable	**0.649**	0.262	0.292	**0.659**	0.195	0.241
Rice	**0.365**	-0.162	-0.052	**0.356**	-0.250	0.021
Tofu	**0.640**	0.122	0.053	**0.660**	0.082	0.022
White vegetable	**0.700**	0.110	0.247	**0.708**	0.050	0.191
Bread	-0.205	**0.368**	0.266	-0.146	0.271	**0.308**
Fruit juice	-0.017	**0.668**	0.146	-0.029	**0.676**	0.142
Milk	0.178	**0.404**	-0.054	0.221	0.292	0.050
Soy milk	0.069	**0.410**	-0.021	0.068	**0.410**	-0.010
Vegetable juice	-0.046	**0.685**	0.061	0.023	**0.719**	0.051
Yogurt	0.244	**0.554**	-0.066	**0.307**	**0.480**	-0.082
Beef/pork	0.100	-0.040	**0.736**	0.148	-0.074	**0.745**
Chicken	0.129	0.035	**0.705**	0.137	0.065	**0.683**
Ham/sausage	0.015	0.047	**0.688**	0.013	0.035	**0.690**

FHMS, Fukushima Health Management Survey. [a] highlight for factor loadings >0.3.

Table S2. Associations between dietary patterns and diabetes mellitus incident risk, 2011–2018 for participants aged 20-89 years without diabetes, cardiovascular diseases, or cancer at baseline, FHMS.

	Dietary pattern	All (n = 19,811)		Men (n = 7046)		Women (n = 12,765)	
	scores	HR	95% CI	HR	95% CI	HR	95% CI
Typical Japanese							
Model 1 [a]	Q1 (lowest)	Ref.	-	Ref.	-	Ref.	-
	Q2	**0.82**	**(0.69, 0.97)**	0.81	(0.64, 1.04)	0.82	(0.65, 1.05)
	Q3	**0.81**	**(0.68, 0.95)**	**0.70**	**(0.55, 0.90)**	0.90	(0.72, 1.14)
	Q4	**0.68**	**(0.57, 0.82)**	**0.77**	**(0.60, 0.99)**	**0.62**	**(0.48, 0.79)**
	P for trend	**<0.001**		**0.043**		**<0.001**	
Model 2 [b]	Q1 (lowest)	Ref.	-	Ref.	-	Ref.	-
	Q2	**0.83**	**(0.70, 0.98)**	0.82	(0.64, 1.04)	0.84	(0.66, 1.07)
	Q3	**0.81**	**(0.68, 0.96)**	**0.69**	**(0.54, 0.89)**	0.93	(0.73, 1.17)
	Q4	**0.71**	**(0.59, 0.84)**	**0.76**	**(0.59, 0.97)**	**0.67**	**(0.52, 0.86)**
	P for trend	**<0.001**		**0.028**		**0.004**	
Model 3 [c]	Q1 (lowest)	Ref.	-	Ref.	-	Ref.	-
	Q2	0.85	(0.72, 1.01)	0.85	(0.66, 1.08)	0.86	(0.67, 1.10)
	Q3	0.85	(0.72, 1.01)	**0.73**	**(0.57, 0.93)**	0.97	(0.76, 1.23)
	Q4	**0.77**	**(0.65, 0.92)**	0.84	(0.65, 1.08)	**0.73**	**(0.56, 0.94)**
	P for trend	**0.008**		0.159		**0.026**	
Juice							
Model 1 [a]	Q1 (lowest)	Ref.	-	Ref.	-	Ref.	-
	Q2	0.99	(0.85, 1.17)	0.93	(0.74, 1.18)	1.05	(0.85, 1.32)
	Q3	0.86	(0.73, 1.01)	0.82	(0.65, 1.04)	0.90	(0.71, 1.13)
	Q4	0.95	(0.81, 1.11)	0.92	(0.73, 1.16)	0.98	(0.79, 1.23)
	P for trend	0.354		0.457		0.661	
Model 2 [b]	Q1 (lowest)	Ref.	-	Ref.	-	Ref.	-
	Q2	0.99	(0.84, 1.16)	0.92	(0.73, 1.17)	1.06	(0.85, 1.32)
	Q3	0.84	(0.72, 1.00)	0.81	(0.64, 1.03)	0.89	(0.70, 1.12)
	Q4	0.94	(0.80, 1.11)	0.90	(0.72, 1.13)	1.02	(0.81, 1.27)
	P for trend	0.333		0.349		0.863	
Model 3 [c]	Q1 (lowest)	Ref.	-	Ref.	-	Ref.	-
	Q2	1.00	(0.85, 1.17)	0.94	(0.74, 1.19)	1.06	(0.84, 1.32)
	Q3	0.87	(0.73, 1.02)	0.84	(0.66, 1.07)	0.89	(0.70, 1.12)
	Q4	0.99	(0.84, 1.17)	0.95	(0.75, 1.21)	1.04	(0.83, 1.31)
	P for trend	0.735		0.679		0.939	
Meat							
Model 1 [a]	Q1 (lowest)	Ref.	-	Ref.	-	Ref.	-
	Q2	1.10	(0.95, 1.28)	1.04	(0.83, 1.29)	1.17	(0.96, 1.44)
	Q3	0.87	(0.74, 1.03)	0.90	(0.71, 1.14)	0.85	(0.67, 1.07)
	Q4	0.99	(0.84, 1.17)	0.99	(0.78, 1.27)	0.99	(0.79, 1.24)
	P for trend	0.431		0.734		0.458	
Model 2 [b]	Q1 (lowest)	Ref.	-	Ref.	-	Ref.	-
	Q2	1.10	(0.94, 1.27)	1.03	(0.83, 1.28)	1.18	(0.96, 1.45)

	Q3	0.89	(0.75, 1.05)	0.92	(0.72, 1.17)	0.85	(0.68, 1.08)
	Q4	1.01	(0.85, 1.19)	1.01	(0.79, 1.29)	1.00	(0.80, 1.26)
	P for trend	0.599		0.881		0.527	
Model 3 [c]	Q1 (lowest)	Ref.	-	Ref.	-	Ref.	-
	Q2	1.11	(0.95, 1.29)	1.03	(0.83, 1.28)	1.20	(0.97, 1.47)
	Q3	0.89	(0.76, 1.05)	0.91	(0.72, 1.16)	0.87	(0.69, 1.09)
	Q4	1.04	(0.88, 1.22)	1.01	(0.79, 1.30)	1.04	(0.83, 1.31)
	P for trend	0.828		0.908		0.768	

[a.] Adjusted for age (continuous) and sex; [b.] further adjusted for body mass index (<23, 23-<25, ≥25 kg/m^2); [c.] further adjusted for smoking (no, ever), drinking (no, yes), education level (<, ≥vocational university), physical activity (<two, ≥two times/wk), Kessler Psychological Distress Scale (<13, ≥13), change of residence (no, yes), hypertension (no, yes), high-density lipoprotein cholesterol <40 mg/L (no, yes), low-density lipoprotein cholesterol ≥140 mg/L (no, yes), and triglyceride ≥150 mg/L (no, yes). HR, hazard ratio; CI, confidential interval; FHMS, Fukushima Health Management Survey.

Table S3. Associations between dietary patterns and diabetes mellitus incident risk, 2011–2018 for participants aged 40-74 years without diabetes, cardiovascular diseases, or cancer at baseline, FHMS.

	Dietary pattern scores	All (n = 13,623) HR	95% CI	Men (n = 4942) HR	95% CI	Women (n = 8681) HR	95% CI
Typical Japanese							
Model 1[a]	Q1 (lowest)	Ref.	-	Ref.	-	Ref.	-
	Q2	0.86	(0.72, 1.04)	0.82	(0.63, 1.06)	0.91	(0.70, 1.18)
	Q3	0.89	(0.74, 1.06)	**0.72**	**(0.55, 0.94)**	1.06	(0.82, 1.35)
	Q4	**0.72**	**(0.59, 0.87)**	0.80	(0.61, 1.05)	**0.64**	**(0.49, 0.85)**
	P for trend	**0.002**		0.093		**0.006**	
Model 2[b]	Q1 (lowest)	Ref.	-	Ref.	-	Ref.	-
	Q2	0.87	(0.73, 1.05)	0.82	(0.63, 1.06)	0.93	(0.72, 1.20)
	Q3	0.88	(0.74, 1.06)	**0.70**	**(0.53, 0.92)**	1.08	(0.84, 1.38)
	Q4	**0.74**	**(0.61, 0.89)**	0.78	(0.59, 1.02)	**0.70**	**(0.53, 0.93)**
	P for trend	**0.003**		0.055		**0.035**	
Model 3[c]	Q1 (lowest)	Ref.	-	Ref.	-	Ref.	-
	Q2	0.89	(0.74, 1.07)	0.86	(0.66, 1.11)	0.94	(0.73, 1.22)
	Q3	0.93	(0.77, 1.11)	**0.75**	**(0.57, 0.98)**	1.11	(0.87, 1.43)
	Q4	**0.79**	**(0.65, 0.96)**	0.85	(0.65, 1.12)	**0.75**	**(0.57, 0.99)**
	P for trend	**0.032**		0.211		0.106	
Juice							
Model 1[a]	Q1 (lowest)	Ref.	-	Ref.	-	Ref.	-
	Q2	0.89	(0.74, 1.06)	0.87	(0.66, 1.13)	0.91	(0.71, 1.16)
	Q3	0.81	(0.68, 0.97)	0.85	(0.66, 1.11)	0.77	(0.60, 1.00)
	Q4	0.94	(0.79, 1.12)	0.95	(0.74, 1.23)	0.93	(0.73, 1.19)
	P for trend	0.559		0.871		0.57	
Model 2[b]	Q1 (lowest)	Ref.	-	Ref.	-	Ref.	-
	Q2	0.87	(0.73, 1.05)	0.85	(0.66, 1.11)	0.89	(0.70, 1.14)
	Q3	0.80	(0.66, 0.96)	0.83	(0.64, 1.08)	0.77	(0.60, 1.00)
	Q4	0.93	(0.78, 1.11)	0.92	(0.72, 1.19)	0.96	(0.75, 1.23)
	P for trend	0.502		0.68		0.778	
Model 3[c]	Q1 (lowest)	Ref.	-	Ref.	-	Ref.	-
	Q2	0.88	(0.73, 1.05)	0.87	(0.66, 1.13)	0.88	(0.69, 1.14)
	Q3	0.82	(0.68, 0.98)	0.87	(0.67, 1.14)	0.76	(0.59, 0.99)
	Q4	0.97	(0.81, 1.16)	0.98	(0.75, 1.27)	0.97	(0.76, 1.24)
	P for trend	0.879		0.957		0.839	
Meat							
Model 1[a]	Q1 (lowest)	Ref.	-	Ref.	-	Ref.	-
	Q2	1.14	(0.96, 1.36)	1.02	(0.80, 1.31)	1.29	(1.01, 1.63)
	Q3	0.90	(0.75, 1.09)	0.87	(0.67, 1.13)	0.95	(0.73, 1.23)
	Q4	0.98	(0.82, 1.18)	0.97	(0.74, 1.26)	1.00	(0.77, 1.30)
	P for trend	0.379		0.642		0.479	
Model 2[b]	Q1 (lowest)	Ref.	-	Ref.	-	Ref.	-
	Q2	1.14	(0.96, 1.36)	1.01	(0.79, 1.30)	1.30	(1.02, 1.65)

	Q3	0.92	(0.76, 1.11)	0.87	(0.67, 1.14)	0.96	(0.74, 1.25)
	Q4	1.00	(0.83, 1.20)	0.98	(0.75, 1.28)	1.01	(0.78, 1.32)
	P for trend	0.522		0.749		0.545	
Model 3[c]	Q1 (lowest)	Ref.	-	Ref.	-	Ref.	-
	Q2	1.16	(0.97, 1.38)	1.02	(0.79, 1.30)	1.32	(1.03, 1.67)
	Q3	0.94	(0.78, 1.14)	0.89	(0.68, 1.16)	0.99	(0.76, 1.28)
	Q4	1.01	(0.84, 1.22)	0.97	(0.75, 1.27)	1.04	(0.80, 1.35)
	P for trend	0.626		0.696		0.678	

[a.] Adjusted for age (continuous) and sex; [b.] further adjusted for body mass index (<23, 23-<25, ≥25 kg/m²); [c.] further adjusted for smoking (no, ever), drinking (no, yes), education level (<, ≥vocational university), physical activity (<two, ≥two times/wk), Kessler Psychological Distress Scale (<13, ≥13), change of residence (no, yes), hypertension (no, yes), high-density lipoprotein cholesterol <40 mg/L (no, yes), low-density lipoprotein cholesterol ≥140 mg/L (no, yes), and triglyceride ≥150 mg/L (no, yes). HR, hazard ratio; CI, confidential interval; FHMS, Fukushima Health Management Survey.

Table S4. Associations between dietary patterns and diabetes mellitus incident risk with assessment of changes in pattern scores between 2011 and 2013, FHMS.

	Dietary pattern scores	All (n = 22,740)		Men (n = 8465)		Women (n = 14,275)	
		HR	95% CI	HR	95% CI	HR	95% CI
Typical Japanese							
Model 1[a]	Q1 (lowest)	Ref.	-	Ref.	-	Ref.	-
	Q2	**0.79**	**(0.68, 0.93)**	**0.77**	**(0.62, 0.95)**	0.83	(0.66, 1.03)
	Q3	**0.80**	**(0.68, 0.94)**	**0.70**	**(0.56, 0.88)**	0.92	(0.74, 1.15)
	Q4	**0.73**	**(0.61, 0.86)**	**0.76**	**(0.60, 0.95)**	**0.71**	**(0.56, 0.91)**
	P for trend	**<0.001**		**0.05**		**<0.001**	
Model 2[b]	Q1 (lowest)	Ref.	-	Ref.	-	Ref.	-
	Q2	**0.81**	**(0.69, 0.94)**	**0.78**	**(0.63, 0.96)**	0.84	(0.67, 1.05)
	Q3	**0.81**	**(0.69, 0.94)**	**0.70**	**(0.56, 0.88)**	0.93	(0.74, 1.16)
	Q4	**0.75**	**(0.63, 0.88)**	**0.75**	**(0.60, 0.95)**	**0.75**	**(0.59, 0.96)**
	P for trend	**0.002**		**0.026**		**0.042**	
Model 3[c]	Q1 (lowest)	Ref.	-	Ref.	-	Ref.	-
	Q2	**0.82**	**(0.70, 0.96)**	**0.79**	**(0.63, 0.98)**	0.84	(0.67, 1.06)
	Q3	**0.83**	**(0.71, 0.97)**	**0.71**	**(0.56, 0.89)**	0.95	(0.76, 1.19)
	Q4	**0.79**	**(0.67, 0.94)**	0.80	(0.63, 1.01)	0.79	(0.62, 1.01)
	P for trend	**0.015**		0.078		0.12	
Juice							
Model 1[a]	Q1 (lowest)	Ref.	-	Ref.	-	Ref.	-
	Q2	1.02	(0.88, 1.19)	1.04	(0.84, 1.28)	1.01	(0.83, 1.25)
	Q3	0.92	(0.79, 1.08)	0.99	(0.80, 1.23)	0.87	(0.70, 1.09)
	Q4	1.00	(0.84, 1.18)	1.00	(0.79, 1.26)	1.00	(0.80, 1.25)
	P for trend	0.784		0.695		0.38	
Model 2[b]	Q1 (lowest)	Ref.	-	Ref.	-	Ref.	-
	Q2	1.01	(0.87, 1.17)	1.02	(0.82, 1.25)	1.02	(0.83, 1.25)
	Q3	0.91	(0.78, 1.06)	0.96	(0.77, 1.19)	0.87	(0.70, 1.09)
	Q4	0.99	(0.85, 1.16)	0.97	(0.77, 1.22)	1.04	(0.83, 1.30)
	P for trend	0.659		0.55		0.904	
Model 3[c]	Q1 (lowest)	Ref.	-	Ref.	-	Ref.	-
	Q2	1.02	(0.88, 1.18)	1.03	(0.83, 1.27)	1.01	(0.82, 1.24)
	Q3	0.92	(0.79, 1.07)	0.98	(0.78, 1.21)	0.86	(0.69, 1.07)
	Q4	1.03	(0.87, 1.21)	1.00	(0.79, 1.27)	1.06	(0.85, 1.33)
	P for trend	0.88		0.935		0.698	

Meat							
Model 1[a]	Q1 (lowest)	Ref.	-	Ref.	-	Ref.	-
	Q2	1.14	(0.99, 1.31)	1.12	(0.93, 1.36)	1.16	(0.96, 1.41)
	Q3	0.89	(0.77, 1.04)	0.88	(0.70, 1.09)	0.92	(0.74, 1.14)
	Q4	1.01	(0.86, 1.18)	1.02	(0.82, 1.27)	1.01	(0.81, 1.26)
	P for trend	0.489		0.885		0.289	
Model 2[b]	Q1 (lowest)	Ref.	-	Ref.	-	Ref.	-
	Q2	1.13	(0.99, 1.30)	1.11	(0.91, 1.35)	1.17	(0.96, 1.42)
	Q3	0.91	(0.78, 1.06)	0.89	(0.72, 1.11)	0.92	(0.75, 1.14)
	Q4	1.04	(0.88, 1.21)	1.05	(0.84, 1.31)	1.03	(0.83, 1.29)
	P for trend	0.771		0.934		0.79	
Model 3[c]	Q1 (lowest)	Ref.	-	Ref.	-	Ref.	-
	Q2	1.14	(0.99, 1.30)	1.10	(0.90, 1.33)	1.19	(0.98, 1.44)
	Q3	0.92	(0.79, 1.07)	0.88	(0.71, 1.09)	0.95	(0.76, 1.17)
	Q4	1.06	(0.90, 1.24)	1.04	(0.83, 1.30)	1.08	(0.86, 1.35)
	P for trend	0.992		0.904		0.894	

[a.] Adjusted for age (continuous), sex, changes of dietary pattern score between 2012 and 2011 (decrease, increase, missing), and changes of dietary pattern score between 2013 and 2011 (decrease, increase, missing); [b.] further adjusted for body mass index (<23, 23-<25, ≥25 kg/m^2); [c.] further adjusted for smoking (no, ever), drinking (no, yes), education level (<, ≥vocational university), physical activity (<two, ≥two times/wk), Kessler Psychological Distress Scale (<13, ≥13), change of residence (no, yes), hypertension (no, yes), high-density lipoprotein cholesterol <40 mg/L (no, yes), low-density lipoprotein cholesterol ≥140 mg/L (no, yes), and triglyceride ≥150 mg/L (no, yes). HR, hazard ratio; CI, confidential interval; FHMS, Fukushima Health Management Survey.
